# Reliability of the velocity achieved during the last repetition of sets to failure and its association with the velocity of the 1-repetition maximum

**DOI:** 10.7717/peerj.8760

**Published:** 2020-03-11

**Authors:** Amador García-Ramos, Danica Janicijevic, Jorge M. González-Hernández, Justin W.L. Keogh, Jonathon Weakley

**Affiliations:** 1 Department of Sports Sciences and Physical Conditioning, Universidad Católica de la Santísima Concepción, Concepción, Chile; 2 Department of Physical Education and Sport, Faculty of Sport Sciences, University of Granada, Granada, Spain; 3 Faculty of Sport and Physical Education, The Research Centre, University of Belgrade, Belgrade, Serbia; 4 Faculty of Health Science, Universidad Europea de Canarias, La Orotava, Tenerife, Spain; 5 Faculty of Health Sciences and Medicine, Bond University, Gold Coast, QLD, Australia; 6 Sports Performance Research Centre New Zealand, AUT University, Auckland, New Zealand; 7 Cluster for Health Improvement, Faculty of Science, Health, Education and Engineering, University of the Sunshine Coast, Gold Coast, QLD, Australia; 8 Kasturba Medical College, Manipal Academy of Higher Education, Manipal, Karnataka, India; 9 School of Behavioural and Health Sciences, Australian Campus University, Brisbane, QLD, Australia; 10 Carnegie Applied Rugby Research (CARR) centre, Institute for Sport, Physical Activity and Leisure, Leeds Beckett University, Leeds, UK

**Keywords:** Bench press, Linear position transducer, Minimal velocity threshold, Strength testing, Velocity-based training

## Abstract

**Background:**

This study aimed to determine the reliability of the velocity achieved during the last repetition of sets to failure (*V*_last_) and the association of *V*_last_ with the velocity of the 1-repetition maximum (*V*_1RM_) during the paused and touch-and-go bench press (BP) exercises performed in a Smith machine.

**Methods:**

A total of 96 healthy men participated in this study that consisted of two testing sessions. A single BP variant (paused BP or touch-and-go BP) was evaluated on each session in a randomized order. Each session consisted of an incremental loading test until reaching the 1RM, followed by two sets of repetitions to failure against a load ranging from 75% to 90% of 1RM.

**Results:**

The reliability of *V*_last_ was unacceptable for both BP variants (CV > 18.3%, ICC < 0.60). The correlations between *V*_1RM_ and *V*_last_ were small for the paused BP (*r* = 0.18) and moderate for the touch-and-go BP (*r* = 0.37).

**Conclusions:**

Although these results suggest that *V*_last_ could be a better indicator of the minimal velocity threshold than *V*_1RM_, the low reliability of *V*_last_ and the similar values of *V*_last_ for both BP variants suggest that a standard *V*_1RM_ should be used to estimate the 1RM from the individualized load-velocity relationship.

## Introduction

Movement velocity has been proposed as an accurate variable for estimating the 1-repetition maximum (1RM) during a number of resistance training exercises (García-Ramos & Jaric, 2018; McBurnie et al., 2019). Early studies proposed generalized load-velocity (L-V) relationship equations to estimate the percentage of 1RM from the velocity value recorded against a submaximal load lifted with maximal effort (González-Badillo & Sánchez-Medina, 2010). The basic premise of generalized L-V relationship equations is that a given velocity represents the same percentage of 1RM for all individuals. However, this premise has been refuted in subsequent studies which have shown a greater accuracy in the estimation of the percentage of 1RM using individualized L-V relationships (García-Ramos & Jaric, 2018; García-Ramos et al., 2018a; McBurnie et al., 2019). This is also demonstrated by the between-subject variability in the velocity associated with a given percentage of 1RM being higher than the within-subject variability (Pestaña-Melero et al., 2018). On the basis of these results, an increasing number of studies have recently been conducted to refine the testing procedure of the individualized L-V relationship.

Assessment of individualized L-V relationships requires the recording of movement velocity against at least two submaximal loads and subsequently, the 1RM can be estimated through a linear regression as the load associated with the velocity of the 1RM (*V*_1RM_ or minimal velocity threshold) (García-Ramos & Jaric, 2018; García-Ramos et al., 2018a; McBurnie et al., 2019). However, one of the challenges associated with the use of individualized L-V relationships for predicting the 1RM is how to select the minimal velocity threshold. Previous studies have selected the minimal velocity threshold as either the individualized *V*_1RM_ (Banyard, Nosaka & Haff, 2017; Ruf, Chery & Taylor, 2018) or a general *V*_1RM_ for all subjects (García-Ramos et al., 2018a, 2019a). The assessment of the individualized *V*_1RM_ is associated with at least two problems: (I) the individual is required to perform a lift against the 1RM load and (II) the individual *V*_1RM_ has been demonstrated to be an unreliable metric for a number of exercises such as the back squat (coefficient of variation (CV) = 22.5%, intraclass correlation coefficient (ICC) = 0.42) (Banyard, Nosaka & Haff, 2017), deadlift (CV = 15.7%, ICC = 0.63) (Ruf, Chery & Taylor, 2018), Smith machine bench press (BP) (CV = 13.9–15.7%, ICC = 0.54–0.64) (Pestaña-Melero et al., 2018), or bench pull (CV = 6.36%, ICC = 0.18) (García-Ramos et al., 2019b). Therefore, it would be of interest to investigate whether the minimal velocity threshold (i.e., velocity value used to estimate the 1RM from the individualized L-V relationship) can be obtained with a higher reliability using other approaches that do not require the individual to perform a lift against the 1RM load.

An alternative approach for determining the minimal velocity threshold could be the assessment of the velocity of the last repetition performed during a set to failure (*V*_last_) (Lake et al., 2017). This approach is supported by the results of Izquierdo et al. (2006) who did not find significant differences between the individual *V*_1RM_ and the *V*_last_ collected against four submaximal loads (60%, 65%, 70% and 75% of 1RM) during the BP and parallel back squat exercises performed in a Smith machine. A recent review has suggested that the accuracy of the individualized L-V relationship for predicting the 1RM is higher for upper-body (e.g., BP or bench pull) compared to lower-body exercises (e.g., squat or deadlift), while the BP is the exercise most explored in velocity-based training research (McBurnie et al., 2019). The two main variants of the BP exercise examined in the scientific literature are the paused BP (a pause is introduced between the eccentric and concentric phases) and the touch-and-go BP (the concentric phase is performed immediately after the eccentric phase) (García-Ramos et al., 2018b; Pallarés et al., 2014; Pérez-Castilla et al., 2018). García-Ramos et al. (2018a) revealed a comparable *V*_1RM_ for the paused BP (0.168 ± 0.026 m/s) and touch-and-go BP (0.178 ± 0.030 m/s) (*P* = 0.232) performed in a Smith machine, while the *V*_1RM_ was poorly related between the BP variants (*r* = −0.11, *P* = 0.554). However, no study has examined the reliability of *V*_last_ or the association between *V*_last_ and *V*_1RM_. Therefore, a comprehensive examination of the behavior of *V*_last_ during the BP exercise is needed to clarify whether *V*_last_ may provide useful information for increasing the accuracy in the estimation of the 1RM through the individualized L-V relationship.

To address the aforementioned gaps in the literature, the main aim of the present study was to assess the reliability of *V*_last_ and the association between *V*_last_ and *V*_1RM_ during the paused and touch-and-go BP exercises performed in a Smith machine. In addition, we aimed to determine the effect of the number of repetitions performed to failure on *V*_last_ and the effect of 1RM strength on *V*_1RM_ and *V*_last_. Our main hypothesis was that, regardless of the BP variant, *V*_last_ would present a low level of reliability and it would be poorly correlated with *V*_1RM_. We also hypothesized that no significant correlations would be observed between *V*_last_ and the number of repetitions performed to failure, while the 1RM strength would be negatively associated with *V*_1RM_ and *V*_last_.

## Materials and Methods

### Participants

Ninety-six healthy men volunteered to participate in this study (age = 20.8 ± 3.4 years (range = 18–38 years); body height = 1.73 ± 0.06 m; body mass = 75.3 ± 15.7 kg; paused BP 1RM = 62.3 ± 17.6 kg; touch-and-go BP 1RM = 66.5 ± 18.4 kg; resistance training experience = 1.3 ± 2.4 years (range = 0–10 years)). Eighty-six participants completed both testing sessions, seven participants only performed the paused BP and three participants only performed the touch-and-go BP. A total of 10 participants only performed one BP variant because they reported not to be interested in attending a second testing session. All participants without resistance training experience were first year sport science students and they were familiarized with the BP exercise during several sessions before the onset of the study. Prior to study initiation, participants were informed of the study procedures and provided written informed consent. Additionally, participants were instructed to avoid any strenuous exercise for the 48 h before each testing session. The study protocol adhered to the tenets of the Declaration of Helsinki and was approved by the Institutional Review Board of the University of Granada (491/CEIH/2018).

### Study design

A randomized crossover design was used to comprehensively examine the relationship between *V*_1RM_ and *V*_last_ during the BP exercise performed in a Smith machine. Participants came to the laboratory on two occasions separated by 72–96 h. A single BP variant (paused BP or touch-and-go BP) was evaluated on each session in a randomized order. Each session consisted of an incremental loading test until reaching the 1RM, followed by two sets of repetitions to failure against a load ranging from the 75% of 1RM to the 90% of 1RM. The same participant performed the two sets against the same load for reliability purposes, while the prescribed relative load (% of 1RM) differed between participants to explore the effect of the number of repetitions performed on *V*_last_. Participants were instructed to perform all repetitions at the maximum possible velocity and the mean concentric velocity (MCV, average velocity value from the start of the concentric phase until the velocity of the barbell was 0 m·s^−1^) of the barbell was recorded with a linear velocity transducer (T-Force System; Ergotech, Murcia, Spain). The two sessions for the same participant were held at the same time of the day (±1 h) to minimize the influence of the circadian rhythm on physical performance.

### Procedures

Both testing sessions began with a standardized warm-up which consisted of 5 min of jogging, dynamic stretching, arm and shoulder mobilization and one set of 10 repetitions with an external load of 20 kg (mass of the unloaded Smith machine barbell) in the tested BP variant. The initial load of the incremental loading test was 20 kg and it was increased in 10 kg until the MCV of the barbell was lower than 0.50 ms^−1^. From that moment, the load was increased in increments of 1–5 kg until the 1RM load was achieved. During the incremental loading test, two repetitions were performed when the MCV was higher than 0.50 ms^−1^ and only one repetition when the MCV was lower than 0.50 ms^−1^. The rest between sets was 3 min when the MCV was higher than 0.50 ms^−1^ and 5 min when the MCV was lower than 0.50 ms^−1^.

Ten minutes after the 1RM assessment, participants performed two sets of repetitions to failure with a load ranging between 75% and 90% of the previously determined 1RM. The two sets were separated by 10 min. Participants performed the two sets with the same load for reliability purposes, but the relative load differed between participants to explore the effect of the number of repetitions performed on *V*_last_. Participants were instructed to perform all repetitions at the maximum possible velocity. Two trained spotters were present during the test for safety reasons and to encourage participants to lift the maximum possible load during the 1RM assessment and to perform the maximum possible number of repetitions during the sets of repetitions to failure. The BP was performed in a Smith machine (Ffittech, Taiwan, China), while a linear velocity transducer, which sampled the velocity of the barbell at 1,000 Hz, was used to collect the MCV of all repetitions. Note that the MCV has been reported to be the most accurate velocity variable for determining the load-velocity relationship during the BP exercise (García-Ramos et al., 2018c). The two BP variants evaluated in the present study are described below:

*Paused BP*: Participants initiated the task holding the barbell with their arms fully extended. They lowered the barbell at a self-selected velocity until the barbell made contact with their chest, waited with the barbell on the chest for 2 s and on the word “Go!” performed a purely concentric action at maximum possible velocity until their arms were fully extended.

*Touch-and-go BP*: Participants initiated the task holding the barbell with their arms fully extended. They were instructed to lower the barbell until it touched the chest and then immediately perform the concentric phase at the maximum possible velocity.

### Statistical analysis

Descriptive data are presented as means and standard deviations. The normal distribution of the data was confirmed by the Shapiro–Wilk test (*P* > 0.05). The reliability of *V*_last_ was assessed through the standard error of measurement (SEM) the CV and the ICC (model 3.1) with the corresponding 95% confidence interval. Acceptable reliability was determined as a CV < 10% and a ICC > 0.70 (Cormack et al., 2008). The association between the variables was quantified by the Pearson’s correlation coefficient (*r*) and the following scale was used to quantify the magnitude of the *r* coefficient: trivial (0.00–0.09) small (0.10–0.29) moderate (0.30–0.49) large (0.50–0.69) very large (0.70–0.89) nearly perfect (0.90–0.99) and perfect (1.00) (Hopkins et al., 2009). Paired sample’s *t* tests, the Cohen’s *d* effect size (ES) and Bland–Altman plots were used to compare the magnitude of the variables. The following scale was used to interpret the magnitude of the ES: trivial (<0.20) small (0.20–0.59) moderate (0.60–1.19) large (1.20–1.99) and very large (≥2.00) (Hopkins et al., 2009). The reliability assessment was performed by means of a custom spreadsheet (Hopkins, 2000), while all other statistical analyses were performed using the software SPSS version 22.0 (SPSS, Chicago, IL, USA). Statistical significance was set at an alpha level of 0.05.

## Results

**Figure 1:**
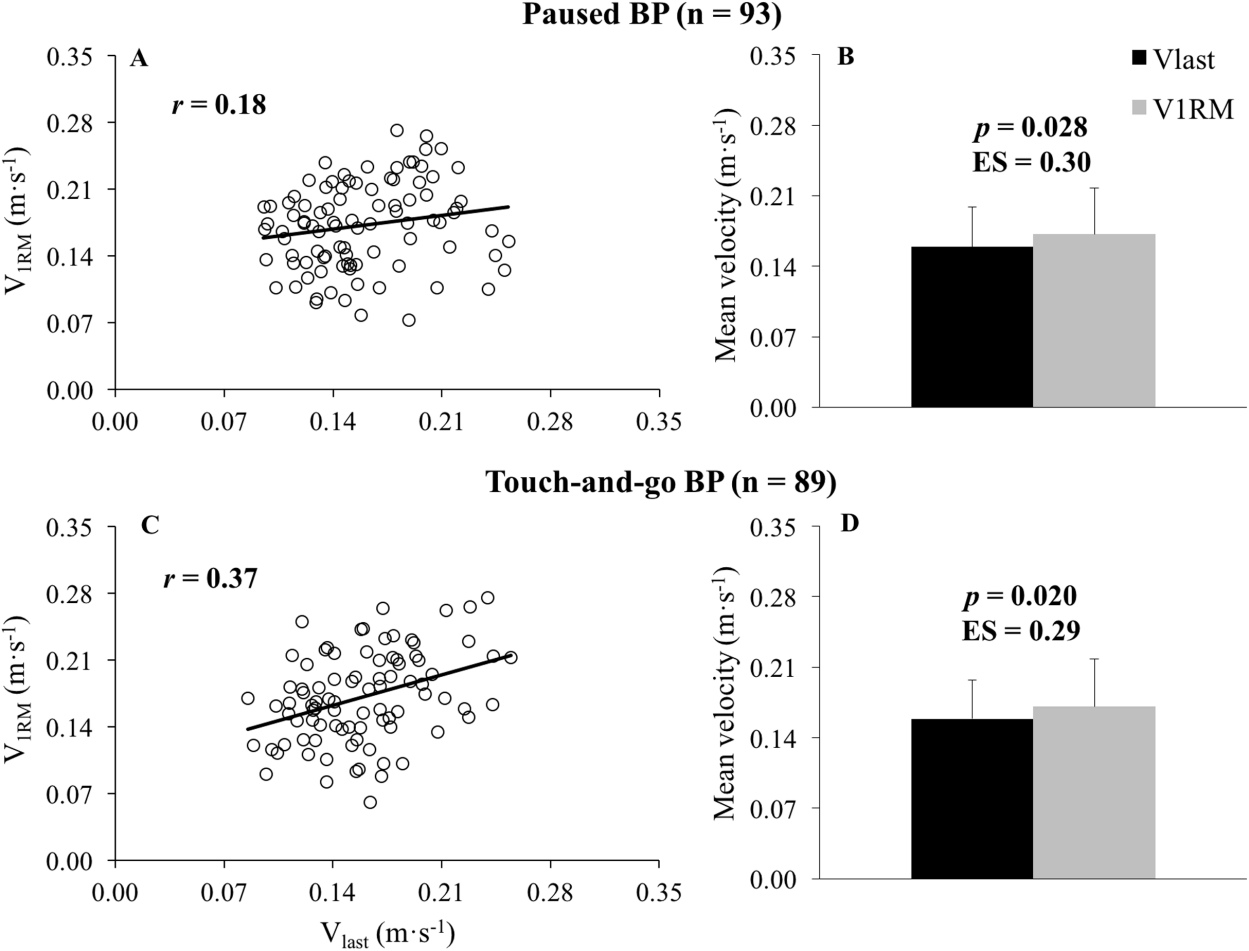
Association and comparison between the velocity of the 1-repetition maximum and the velocity achieved during the last repetition of sets to failure. Association (A and C) and comparison (B and D) between the velocity of the 1-repetition maximum (*V*_1RM_) and the velocity achieved during the last repetition of sets to failure (*V*_last_) during the paused (A and B) and touch-and-go (C and D) bench press (BP) exercises. *r*, Pearson’s correlation coefficient; *p*, *p*-value obtained from paired sample’s *t* tests; ES, Cohen’s *d* effect size ((*V*_1RM_ mean–*V*_last_ mean)/SD both).

**Figure 2:**
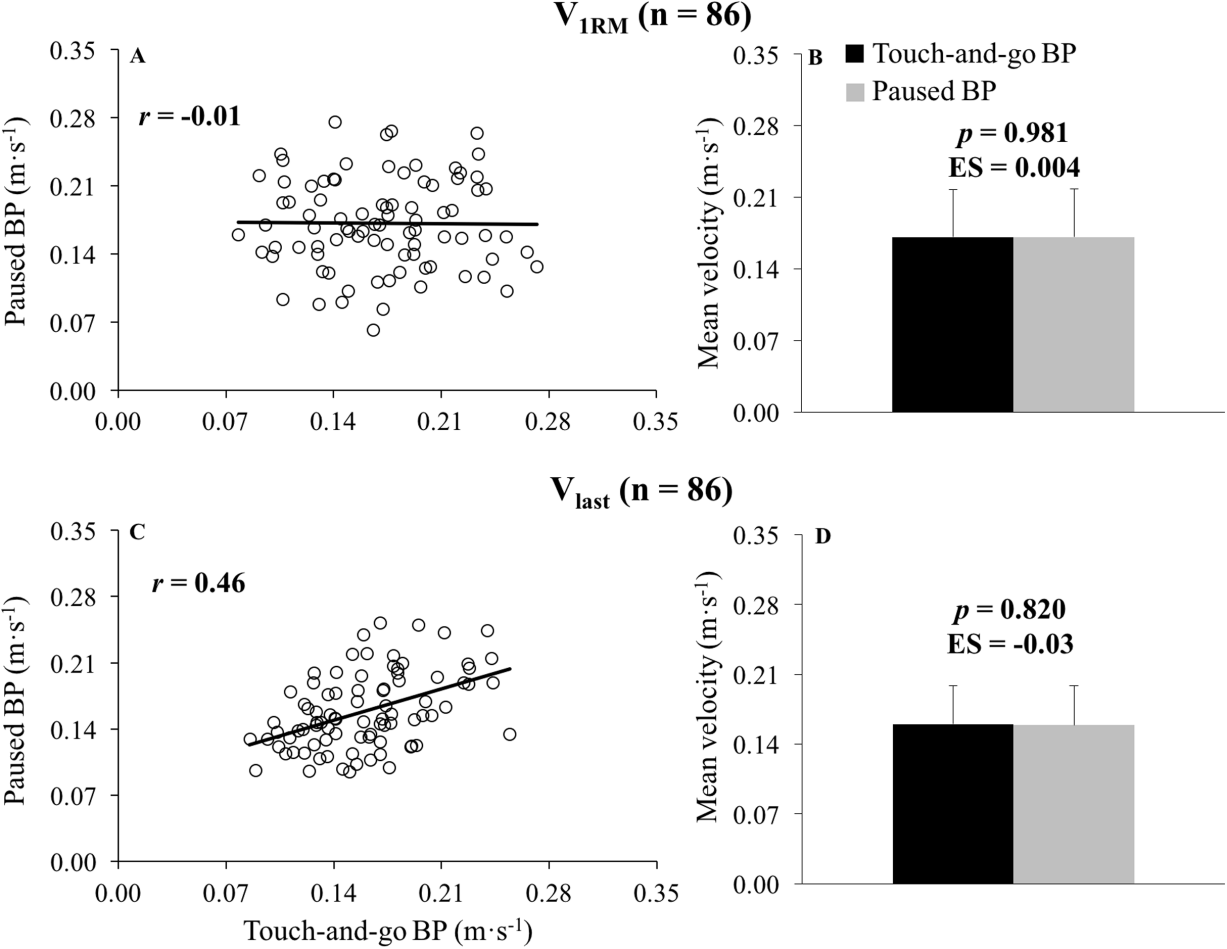
Association and comparison between the paused bench press and the touch-and-go bench press for the velocity of the 1-repetition maximum and the velocity achieved during the last repetition of sets to failure. Association (A and C) and comparison (B and D) between the paused bench press (BP) and the touch-and-go BP for the velocity of the 1-repetition maximum (*V*_1RM_; A and B) and the velocity achieved during the last repetition of sets to failure (*V*_last_; C and D). *r*, Pearson’s correlation coefficient; *p*, *p*-value obtained from paired sample’s *t* tests; ES, Cohen’s *d* effect size ((paused BP mean–touch-and-go BP mean)/SD both).

**Figure 3:**
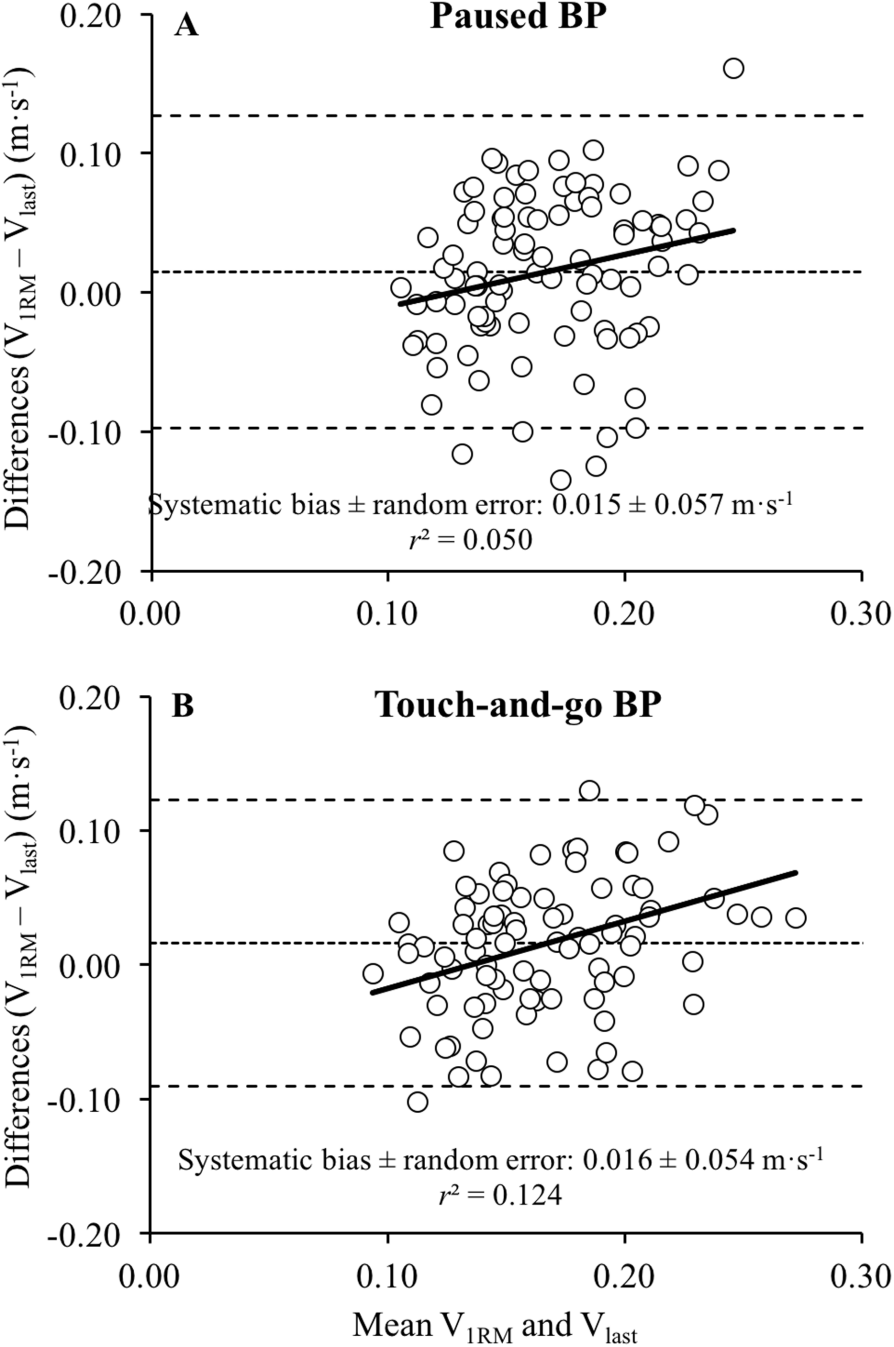
Differences between the velocity of the 1-repetition maximum and the velocity achieved during the last repetition of sets to failure during the paused bench press (BP) and the touch-and-go BP. Bland–Altman plots showing the differences between the velocity of the 1-repetition maximum (*V*_1RM_) and the velocity achieved during the last repetition of sets to failure (*V*_last_) during the paused bench press (BP) (A) and the touch-and-go BP (B). Each plot depicts the systematic bias and 95% limits of agreement (± 1.96 SD; dashed lines), along with the regression line (solid line). The systematic bias ± random error together with the strength of the relationship (*r*^2^) are depicted in each plot.

**Figure 4:**
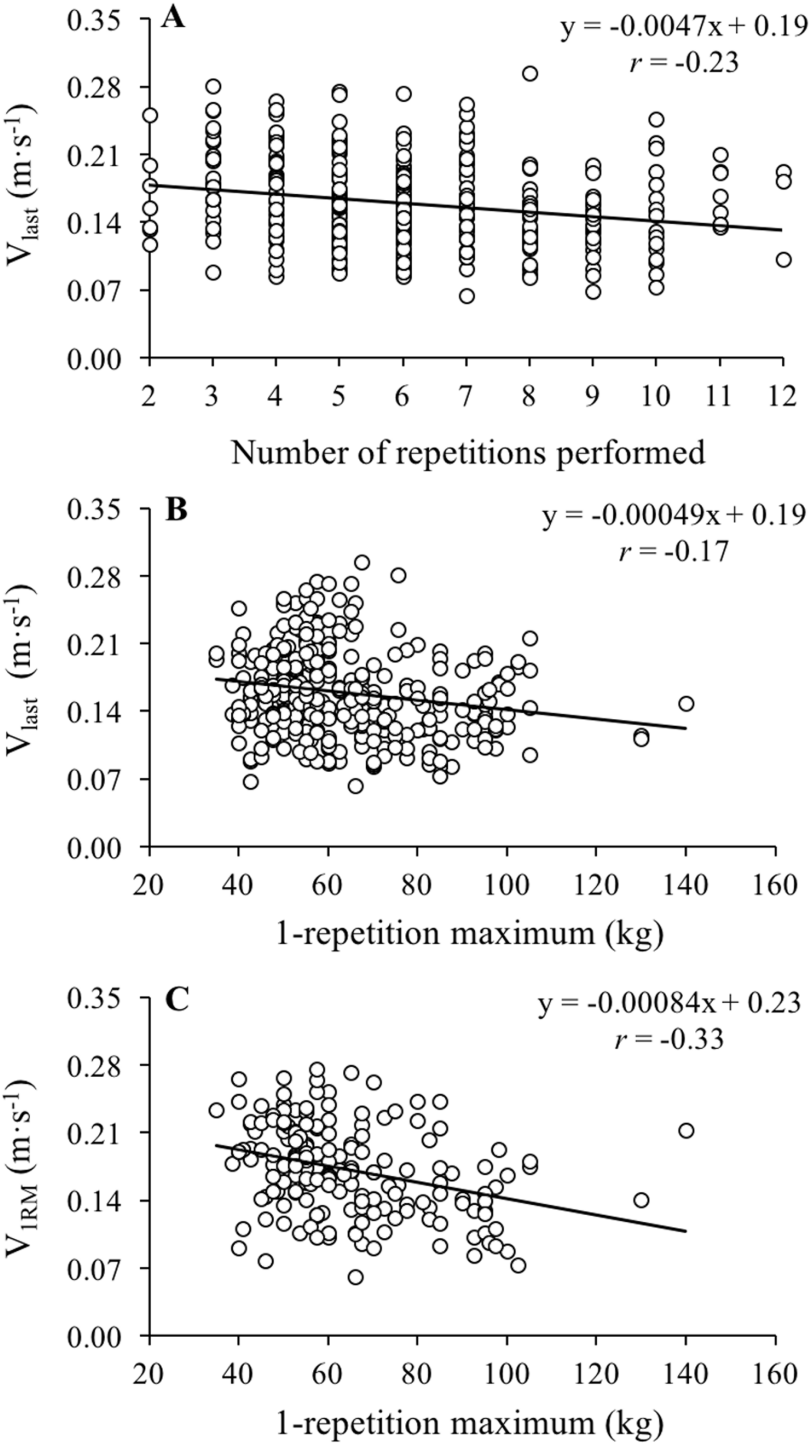
Relationship between the number of repetitions performed to failure and velocity of the last repetition performed during sets of repetitions to failure (*V*_last_), 1-repetition maximum and *V*_last_ and 1-repetition maximum and velocity. Relationship between the number of repetitions performed to failure and *V*_last_ (A) 1-repetition maximum and *V*_last_ (B) and 1-repetition maximum and *V*_1RM_ (C). *V*_last_, velocity of the last repetition performed during sets of repetitions to failure; *V*_1RM_, velocity achieved during the 1-repetition maximum trial. The regression equation and the Pearson’s correlation coefficient (*r*) are depicted.

The reliability of *V*_last_ was unacceptable for both the paused BP (CV = 18.3%, ICC = 0.58) and the touch-and-go BP (CV = 20.4%, ICC = 0.48) (Table 1). The *V*_1RM_ was significantly higher than *V*_last_ during the paused BP (*P* = 0.028, ES = 0.30) and the touch-and-go BP (*P* = 0.020, ES = 0.29), while the magnitude of the correlations between *V*_1RM_ and *V*_last_ was small for the paused BP (*r* = 0.18, *P* = 0.083) and moderate for the touch-and-go BP (*r* = 0.37, *P* < 0.001) (Fig. 1). No significant differences between the BP variants were observed for *V*_1RM_ or *V*_last_ (*P* > 0.80), while the correlations between the BP variants was trivial for *V*_1RM_ (*r* = −0.01, *P* = 0.937) and moderate for *V*_last_ (*r* = 0.46, *P* < 0.001) (Fig. 2). Bland–Altman plots revealed comparable systematic bias (0.015–0.016 m·s^−1^) and random errors (0.054–0.057 m·s^−1^) between *V*_1RM_ and *V*_last_ for both BP variants (Fig. 3). A small and inverse relationship was observed between *V*_last_ and the number of repetitions performed to failure (*r* = −0.23, *P* < 0.001) and the 1RM load (*r* = −0.17, *P* = 0.001), while moderate correlations were observed between *V*_1RM_ and the 1RM load (*r* = −0.33, *P* < 0.001) (Fig. 4). Participants performed the sets of repetitions to failure against an average load of 82.1 ± 3.7% of 1RM and completed 6.9 ± 2.3 repetitions.

**Table 1:** Reliability of mean concentric velocity values achieved during the last repetition of sets to failure during the paused and touch-and-go bench press exercises.

Exercise	Set 1 (m·s^−1^)	Set 2 (m·s^−1^)	*p*	CV (95% CI)	ICC (95% CI)	SEM (m·s^−1^)
Paused BP (*n* = 93)	0.161 (0.045)	0.157 (0.045)	0.303	18.3% [16.0%, 21.4%]	0.58 [0.43, 0.70]	0.029
Touch-and-go BP (*n* = 89)	0.162 (0.046)	0.156 (0.043)	0.266	20.4% [17.8%, 24.0%]	0.48 [0.30, 0.62]	0.032

**Note:**

BP, bench press; *p*, *p*-value; CV, coefficient of variation; ICC, intraclass correlation coefficient; SEM, standard error of measurement; 95% CI, 95% confidence interval.

## Discussion

This study is the first to investigate the reliability of *V*_last_ during the paused and touch-and-go BP exercises and explore the association between *V*_1RM_ and *V*_last_ within and between each BP variant. Additionally, this study determined the effect of the number of repetitions performed to failure on *V*_last_ and the effect of 1RM strength on *V*_1RM_ and *V*_last_. The main findings of the study revealed (I) low reliability for *V*_last_ (II) *V*_last_ was significantly lower than *V*_1RM_ (III) no significant differences between the BP variants for *V*_1RM_ or *V*_last_ (IV) larger associations between the BP variants for *V*_last_ compared to *V*_1RM_ and (V) a negative, albeit weak, association of *V*_last_ with the number of repetitions performed to failure and the 1RM strength. These results suggest that *V*_last_ could be a better indicator of the minimal velocity threshold during the BP exercise than *V*_1RM_ because *V*_last_ was lower than *V*_1RM_ and it demonstrated a stronger relationship between the BP variants. However, the low reliability of *V*_last_, moderate association between the BP variants for *V*_last_ and the lack of differences between the BP variants for the *V*_1RM_ or *V*_last_ suggest that a general *V*_1RM_ could be more appropriate.

Previous studies have found that the magnitude of *V*_last_ is not affected by the load applied during sets of repetitions to failure performed with the BP and back squat exercises (Izquierdo et al., 2006). However, no previous study had examined the reliability of *V*_last_ in any resistance training exercise. Our results confirmed that *V*_last_ is an unreliable metric for the two variants of the BP exercise examined in the present study. The reliability of *V*_last_ (CV = 18.3% and 20.4% for the paused and touch-and-go BP, respectively) was somewhat comparable to the reliability of the MCV associated with the 1RM load reported by Pestaña-Melero et al. (2018) (CV =13.9% and 15.7% for the paused and touch-and-go BP, respectively). Therefore, it seems that neither the *V*_1RM_ nor the *V*_last_ should be used on an individual basis since the within-subject variability of these variables is not meaningfully lower than the variability existing between subjects (Pestaña-Melero et al., 2018).

The generally low correlations between *V*_1RM_ and *V*_last_ for both BP variants (*r* ≤ 0.37) indicates that the value of one variable cannot be inferred from the other. Similarly, as it was previously shown by García-Ramos et al. (2018a) no significant correlations were observed between the two BP variants for *V*_1RM_ (*r* = −0.01). Only the value of *V*_last_ was moderately correlated between the two BP variants (*r* = 0.46), revealing that participants with a higher *V*_last_ in one BP variant also tended to have a higher *V*_last_ in the other BP variant. These results provide additional support for the use of the same minimal velocity threshold (i.e., *V*_1RM_) for all participants when predicting the 1RM from the individualized L-V relationship during the BP exercise performed in a Smith machine. The lack of differences between the BP variants for the magnitudes of *V*_1RM_ and *V*_last_ simplifies this approach because the same minimal velocity threshold could be used for both BP variants. According to the results of this and previous studies, the minimal velocity threshold should be set at 0.17 m·s^−1^ (García-Ramos et al., 2018a; González-Badillo & Sánchez-Medina, 2010; Sánchez-Medina et al., 2014). However, Helms et al. (2017) reported in 15 powerlifters (12 men and 3 women) a lower *V*_1RM_ during the free-weight BP exercise that was performed using a “press” command to simulate a powerlifting competition. Therefore, additional research should be conducted with different variants of the free-weight BP and more trained populations to elucidate whether the minimal velocity threshold recommended in the present study can also be applicable to these conditions.

In the present study we also explored the influence of 1RM strength on the values of *V*_1RM_ and *V*_last_. Based on the lower *V*_1RM_ reported by Helms et al. (2017) in powerlifters during the free-weight BP (0.10 ± 0.04 m·s^−1^) compared to the *V*_1RM_ reported by Loturco et al. (2017) in rugby players and combat athletes (0.17 m·s^−1^), we hypothesized that the 1RM strength would be negatively associated with *V*_1RM_ and *V*_last_. This hypothesis was somewhat supported with a significant, albeit weak, negative correlation between the 1RM load and *V*_1RM_ (*r* = −0.33) and *V*_last_ (*r* = −0.17). This result is also in line with the findings of Ormsbee et al. (2019) who reported a slower *V*_1RM_ for experienced benchers (0.14 ± 0.04 m·s^−1^) compared to novice benchers (0.20 ± 0.05 m·s^−1^). Therefore, although based on these results a slightly lower minimal velocity threshold could be obtained by stronger subjects, the low reliability and low correlations found in the present study for *V*_1RM_ and *V*_last_ suggest that it may not be necessary to modify the minimal velocity threshold based on the 1RM strength values when the BP is performed in a Smith machine. Finally, we also explored the influence of the number of repetitions performed during the sets to failure on *V*_last_ and we observed a relatively weak negative association (*r* = −0.23), indicating that a slightly lower *V*_last_ can be obtained when higher number of repetitions are performed. Therefore, in the case that coaches are interested in determining *V*_last_, we recommended that *V*_last_ is derived from a set to failure of at least five repetitions.

A limitation of the present study is that the 1RM was directly assessed only once for each BP variant and therefore, it was not possible to compare the reliability between *V*_1RM_ and *V*_last_. However, the lower correlations between the BP variants observed in this study for *V*_1RM_ compared to *V*_last_ suggest that the *V*_1RM_ could also be less reliable than *V*_last_. This would corroborate the results of previous studies that have reported a poor reliability for the *V*_1RM_ during the BP (Pestaña-Melero et al., 2018) and other resistance training exercises such as the back squat (Banyard, Nosaka & Haff, 2017), deadlift (Ruf, Chery & Taylor, 2018), or bench pull (García-Ramos et al., 2019b). Another limitation is that the BP was performed in a Smith machine, while the vast majority of athletes use the free-weight BP during their resistance training programs. In the current study, the Smith machine was used to simplify the testing procedure and to improve the reproducibility of velocity readings. Thus, it is plausible that the reliability of *V*_last_ and *V*_1RM_ may be even lower if the BP is performed with free-weights due to the additional control required and horizontal movements characterizing the free weight BP. Finally, the sample of this study was composed of males with a mean of 1.3 years of experience with the BP exercise and therefore, it is possible that the accuracy of *V*_1RM_ and *V*_last_ could increase when testing participants with more experience. Future studies should compare the magnitude of *V*_1RM_ and *V*_last_ as well as the reliability of these variables between individuals with different training backgrounds to shed light on this topic.

## Conclusions

The reliability of *V*_last_ was below the threshold of acceptable reliability for both BP variants. *V*_last_ was always significantly lower than *V*_1RM_, while no significant differences between the BP variants were observed for *V*_1RM_ or *V*_last_. *V*_last_ was more correlated between the BP variants than *V*_1RM_. The correlations between *V*_1RM_ and *V*_last_ ranged from small (paused BP) to moderate (touch-and-go BP). An inverse, but generally weak, association was observed between *V*_last_ and the number of repetitions performed to failure and the 1RM load, as well as between the *V*_1RM_ and the 1RM load. Therefore, even though *V*_last_ could be a more appropriate indicator of the minimal velocity threshold than *V*_1RM_, our results (i.e., low reliability, lack of differences in magnitude and only moderate association between the BP variants) also suggest that a general *V*_1RM_ could be more appropriate to estimate the 1RM during the BP exercise performed in a Smith machine.

## Supplemental Information

10.7717/peerj.8760/supp-1 Supplemental Information 1 Velocity values obtained during 1 repetition maximum and last repetition. CONC-concentric-only bench press; 1RM-one repetition maximum; Reps-number of repetition; CEA-stretch shortening cycle; Aver- average.

## Additional Information and Declarations

### Data Availability

The following information was supplied regarding data availability: The data is available in the Supplemental Files.
